# Regulation of Camphor Metabolism: Induction and Repression of Relevant Monooxygenases in *Pseudomonas putida* NCIMB 10007

**DOI:** 10.3390/microorganisms6020041

**Published:** 2018-05-07

**Authors:** Andrew Willetts, Pamela Masters, Carol Steadman

**Affiliations:** 1 College of Life and Environmental Sciences, University of Exeter, Exeter EX4 4QG, UK; 2 Curnow Consultancies, Helston TR13 9PQ, UK; pammasters@linuxmail.org (P.M.); carol.steadman@mail.com (C.S.)

**Keywords:** cytochrome P450 monooxygenase, diketocamphane monooxygenase: 5-*exo*-hydroxycamphor dehydrogenase, 2-oxo-∆^3^-4,5,5-trimethylcyclopentenylacetic acid monooxygenase, *Pseudomonas putida* NCIMB 10007, (*rac*)-camphor, transcription, rifampicin, actinomycin D

## Abstract

For the first time, the differential rates of synthesis of all the key monooxygenases involved in the catabolism by *Pseudomonas putida* NCIMB 10007 of bicyclic (*rac*)-camphor to ∆^2,5^-3,4,4-trimethylpimelyl-CoA, the first aliphatic pathway intermediate, have been determined to help establish the relevant induction profile of each of the oxygen-dependent enzymes. The efficacy of both relevant substrates and pathway metabolites as inducers has been established. Further, inhibitors with characterised functionality have been used to indicate that the pertinent regulatory controls operate at the level of transcription of the corresponding genes.

## 1. Introduction

**Figure 1:**
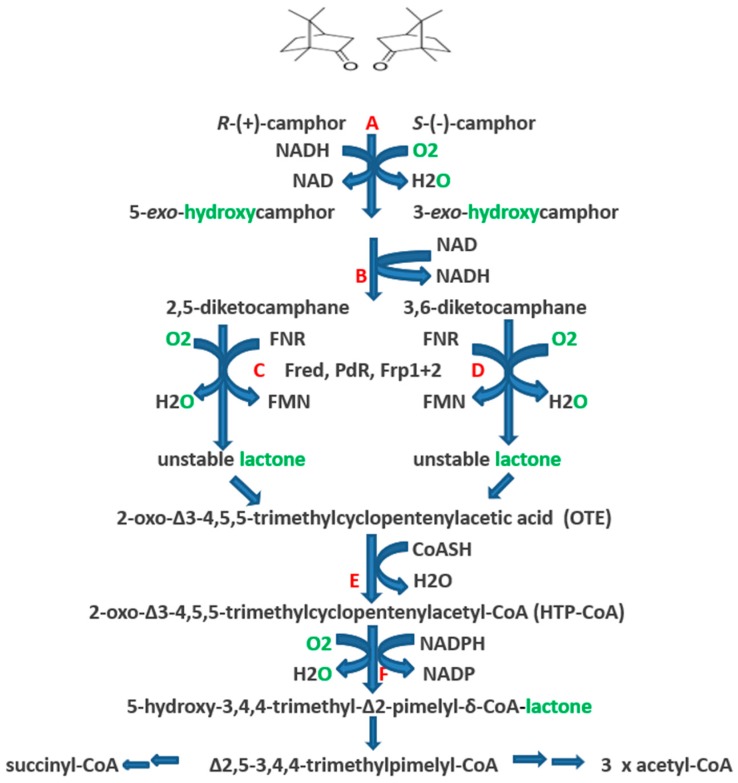
Pathway of (+)- and (−)-camphor degradation in *P. putida* NCIMB 10007. A = cytochrome P450 monooxygenase (*camCAB*): B = *exo*-hydroxycamphor dehydrogenase (*camD*): C = 2,5-diketocamphane 1,2-monooxygenase (*camE_25-1_* + *camE_25-2_*): D = 3,6-diketocamphane 1,6-monooxygenase (*camE_36_*): E = 2-oxo-∆^3^-4,5,5-trimethylcyclopentenylacetyl-CoA synthetase (*camF1* + *F2*): F = 2-oxo-∆^3^-4,5,5-trimethylcyclopentenylacetyl-CoA monooxygenase (*camG*): Fred = 36 kDa chromosome-coded flavin reductase: PdR = putidaredoxin reductase subunit of cytochrome P45O monooxygenase (*camA*): Frp 1 + 2 = chromosome-coded ferric reductases: diatomic oxygen molecules participating in the four monooxygenase-catalysed steps is shown in green, as in each case are the fates of each component oxygen atom.

The identification and partial characterisation of the sequence of catabolic reactions that serves to integrate the monoterpenoid camphor into the TCA cycle in *Pseudomonas putida* NCIMB 10007 (Figure 1) resulted from the pioneering studies initiated by Irwin Gunsalus et al. nearly 60 years ago [1,2]. Although the contribution of genes located on the CAM-plasmid present in the bacterium to the degradation of the bicyclic terpene to ∆^2,5^-3,4,4-trimethylpimelyl-CoA was first recognised by Chakrabarty et al. [3,4], it is only recently [5,6] that it has been possible to assign plasmid-coded proteins acting either as enzymes or redox intermediates for every activity necessary to metabolise both camphor enantiomers to the level of isobutyryl-CoA, a metabolite also generated as an intermediate in the chromosome-coded pathways for the catabolism of pantothenate and the amino acid valine in pseudomonads [7]. 

The key facilitating reactions in the (*rac*)-camphor degradative pathway are catalysed by four monooxygenases. The pathway is initiated by cytochrome P450 monooxygenase (cytP450MO: A = *camA* + *camB* + *camC*), which promotes the initial activation of the bicyclic ring of both enantiomers of camphor equally. The next key oxygen-dependent reactions are catalysed by the enantiocomplementary 2,5-diketocamphane 1,2-monooxygenase (2,5-DKCMO; C = *camE_25-1_* + *camE_25-2_*) and 3,6-diketocamphane 1,6-monooxygenase (3,6-DKCMO; D = *camE_36_*) isoenzymes which serve to cleave the chiral bicyclic diketones generated respectively from the (+)- and (−)-enantiomers of (*rac*)-camphor to the common achiral monocyclic intermediate 2-oxo-∆^3^-4,5,5-trimethylcyclopentenylacetic acid (OTE, originally referred to as Acid 1 [3]). Finally, OTE monooxygenase (OTEMO; F = *camG*) generates 5-hydroxy-3,4,4-trimethyl-∆^2^-pimelyl-CoA-δ-lactone (HTP-CoA), an unstable lactone that undergoes spontaneous hydrolytic cleavage to yield ∆^2,5^-3,4,4-trimethylpimelyl-CoA, a carboxylic acid ester which is notable for being the first aliphatic intermediate in the camphor degradation pathway. Unlike cytP450MO and OTEMO which both function exclusively as CAM-plasmid coded activities, the diketocamphane monooxygenases (DKCMO) are reduced flavin mononucleotide (FNR)-dependent two-component monooxygenases (fd-TCMOs) which are able to deploy not only CAM-plasmid coded putidaredoxin reductase (PdR) but alternatively three chromosome-coded enzymes, the dimeric flavin reductase [FR] Fred [5] and the closely related ferric reductases Frp1 and FRp2, as alternative requisite sources of FNR [6,8].

Preliminary studies that focused mainly on the induction of various epimeric hydroxycamphor dehydrogenases (HOcamDHs) but only superficially on the DKCMOs obtained some evidence for elements of regulatory controls that implicated product as well as substrate effectors [9], allied to relaxed effector specificity [10], although the relevant molecular modes of action were not identified. This interim conclusion was supported by a more recent complementary study of progressive changes in the levels of a number of key camphor-degrading enzymes throughout diauxic growth of *P. putida* NCIMB 10007 on a composite succinate-camphor medium [8]. However, to date there have been no comprehensive studies of either the progress of the relevant key initial regulatory events triggered by competent effector compounds acting under ‘gratuitous conditions’ [10,11,12], or the molecular mode(s) of action that influence the levels of the key monooxygenases and none that take account of the potential of the isoenzymic DKCMOs to engage in alternative flavin-dependent redox transfers involving either CAM-plasmid coded (PdR) or chromosome-coded (Fred, Frp1 and 2) FNR-generating activities. A number of different experimental approaches used to address these deficiencies have identified several relevant transcription-based regulatory controls and these studies are the subject of the present paper.

## 2. Materials and Methods

### 2.1. Bacterial Strains, Culture Maintenance and Growth Conditions

*P. putida* NCIMB 10007 (ATCC 17453; C1-B; PpG1) was maintained on nutrient agar slopes as described previously [13]. All media used were prepared using the same mineral salt base [14]; the principal carbon source sodium succinate was sterilised separately and added aseptically and all potential effector compounds (inducers, inducer-substrates, corepressors) were added by filter sterilisation. The stock culture used to test any effector compound was initially grown aerobically at 30 °C on a rotary shaker up to mid log phase (A_500_nm 1.6) in 2 L of succinate minimal medium (5 mM), then harvested and used as the inoculum for the test itself which was subsequently carried out in 500 mL of fresh succinate minimal medium (2.5 mM). The 500 mL freshly inoculated culture was grown on aerobically at 30 °C on a rotary shaker and subsequent growth both before and after the addition of an effector compound measured turbimetrically using the same instrument (Bausch and Lomb Spectronic 20) that had been used to prepare a calibration curve relating the corresponding A_500_nm values to equivalent dry weight values for these growth conditions. An equivalent protocol was used to study growth under anaerobic conditions except that the recovered initial inoculum was made up to 500 mL with fresh succinate minimal medium (2.5 mM) supplemented with 10 mM NaNO_3_ and then flushed with argon for 15 min prior to containment in a sealed 500 mL medium bottle fitted with a rubber septum, enabling samples to be removed subsequently for analysis. 

Potential effector compounds (5 mM, unless otherwise stated) were added aseptically to cultures growing on 2.5 mM succinate minimal medium in late log phase of growth (A_500_nm = 0.7) when the remaining succinate level was <0.45 mM: as a preliminary step, two pre-addition samples (20 mL) of the succinate-grown culture were taken (−30 min and −15 min) to enable some relevant parameters to be monitored. As was noted previously when using a similar experimental protocol with succinate-grown cells [9,15], although the cells continued to grow on the diminishingly low level of residual succinate, known camphor pathway intermediates were detected in the sample taken 15 min after adding (*rac*)-camphor as the effector compound and progressively accumulated in the medium thereafter. Consequently, until the succinate is completely depleted (75–90 min after adding each of the tested effector compound), any recorded changes in relevant enzyme titres will therefore have occurred under ‘gratuitous conditions’ [10,11,12]. When testing the effect of the co-addition of rifampicin and actinomycin D, the antibiotics were added aseptically as an aqueous solution (2 μM and 5 μM respectively) at the same time as the effector compound.

The efficacy of each effector compound confirmed as an inducer was further checked as a potential growth substrate by monitoring the change in A_500_nm of *P. putida* NCIMB 10007 after inoculation into basic minimal medium containing 3 mM of the confirmed inducer as the sole carbon source. In addition, a number of compounds structurally related to either bicyclic camphor or monocyclic OTE were tested, as well as a number of 17-keto steroids introduced into the minimal medium at 2 mM because of limiting solubility considerations.

### 2.2. Extract Preparation

Culture samples (20 mL), taken at timed intervals both before (−30 min and −15 min) and after (routinely 0–135 min, +/− additional samples at 180 min and 210 min) the addition of an effector compound, were harvested by centrifugation (10,000× *g* for 15 min at 5 °C), washed with an equal volume of cold Tris-HCl buffer (0.1 M, pH 7.0) and then re-centrifuged. The recovered cells were evenly suspended in 7.5 mL of the same buffer and subsequently sonicated (Soniprep 150: MSE Ltd., Heathfield, East Sussex TN21 8DB, UK) in ice for 3 × 2 min. The resulting homogenate was centrifuged (20,000× *g* for 15 min at 5 °C) to remove the cell debris prior to subjecting the supernatant to three successive rounds of ultrafiltration (15,000× *g* for 15 min at 5 °C) using Amicon centrifugal filters with molecular weight cut-off values of 100 kDa, followed by 50 kDa and finally 30 kDa. This regime generated four fractions (>100 kDa, 100–50 kDa, 50–30 kDa and <30 kDa) which were then selectively assayed for OTEMO (MW 118 kDa, >100 kDa fraction), cytP450MO (MW 98 kDa, typically 90% of the total titre was in the 100–50 kDa fraction), 3,6-DKCMO (MW 85 kDa, 100–50 kDa fraction), 2,5-DKCMO (MW *camE_25-2_* 65 kDa, MW *camE_25-1_* 60 kDa, 100–50 kDa fraction), 5-*exo*-HOcamDH (MW 36 kDa, 50–30 kDa fraction), Fred (MW 36 kDa, 50–30 kDa fraction) and Frp1 plus Frp2 (MWs 27.0 kDa and 28.5 kDa respectively, <30 kDa fraction).

### 2.3. Enzyme Assays

All enzyme assays were conducted with aliquots of the relevant size-dependent fraction by the following previously reported fully described well established methods: OTEMO [16]; cytP450MO [17]; 2,5- and 3,6-DKCMOs [6,8]; 5-*exo*-HOcamDH [10]; Fred [8]; combined Frp1 plus Frp2 activity [18]. 

### 2.4. Chemical Analyses

GC analyses of camphor, camphor metabolites and related bicyclic and monocyclic compounds were performed using both BP1 and Lipodex D capillary columns by previous fully described methods [8,13]. 

### 2.5. Reproducibility

All described procedures were repeated a minimum of 5 times unless otherwise stated with equivalent cultures and partially purified enzyme preparations. As a high degree of consistency (+/− 0–7%) was recorded both between replicate cultures and replicate measurements, all results are presented as averaged values.

### 2.6. Chemicals and General Procedures

Unless otherwise stated, all chemicals, enzymes and reagents were purchased from Sigma-Aldrich (St Louis, MO, USA) or Thermo Fisher Scientific (Loughborough, UK) and were used without further purification. The camphor metabolites 5-*exo*-hydroxycamphor, 3-*exo*-hydroxycamphor, 2,5-diketocamphane (2,5-DKC), 3,6-diketocamphane (3,6-DKC) and OTE were extracted from large (5 L) samples of spent (*rac*)-camphor-based medium using previously described acid-base separation methods [1,10].

## 3. Results

### 3.1. Differential Rates of Synthesis of Key Enzyme Activities Induced by (Rac)-Camphor

**Figure 2:**
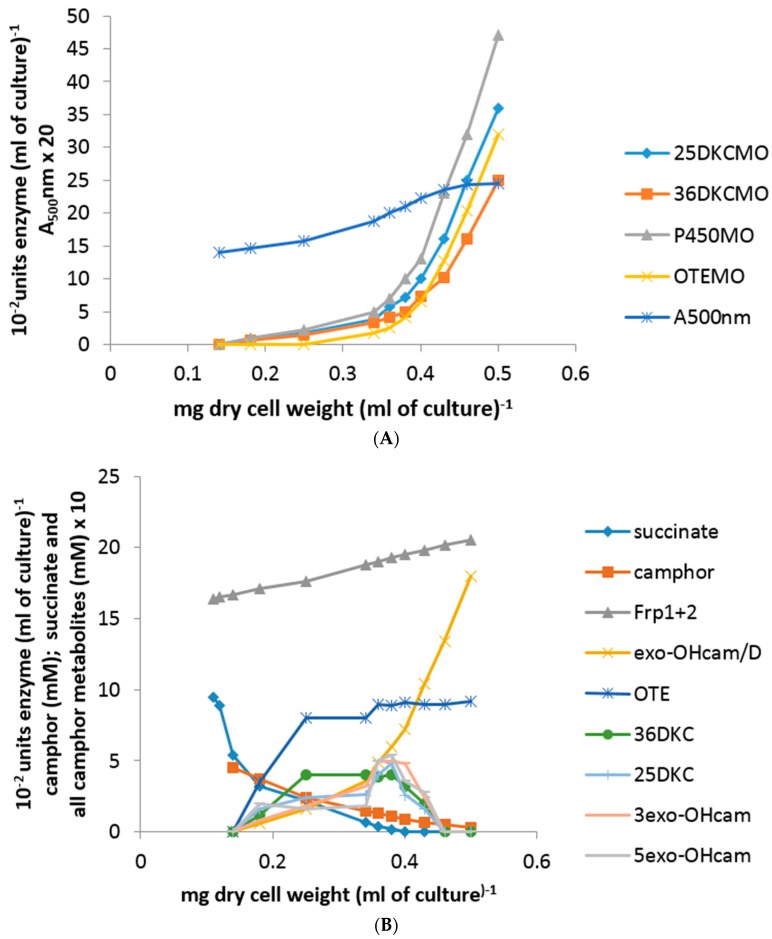
(**A**,**B**) Changes in the optical density (A_500_nm) of *P. putida* NCIMB 10007 following addition of (*rac*)-camphor as inducer-substrate to succinate minimal medium and concomitant changes in the activity of key enzymes and metabolic intermediates of the camphor degradation pathway.

Plotting the total concentration of an enzyme (units enzyme [mL of culture^−1^]) against the culture density (mg dry cell weight [mL of culture^−1^]) generates a so-called differential plot which gives a measure of the rate of synthesis of an enzyme in relation to total cell synthesis [10,11,12]: the slope of such a plot, which corresponds to the specific activity of the enzyme, was termed the differential rate of synthesis by Jacob and Monod [11] and gives a visual representation of the induction rate of the enzyme [19]. Very similar patterns of progressively accelerating differential rates of synthesis of cytP450MO, 5-*exo*-HOcamDH (which is equally active with the corresponding 3-*exo* enantiomer [9,20]) and 2,5-DKCMO were obtained when the activities of these enzymes were measured at every 15 min interval throughout the 135 min following 5 mM (*rac*)-camphor addition to a culture (Figure 2A,B). Significantly, the differential rate of synthesis of 3,6-DKCMO was noticeably slower, which correlated with the consistently lower titre for this enzyme compared to the enantio-complementary 2,5-DKCMO recorded in extensive prior studies with (*rac*)-camphor-grown *P. putida* NCIMB 10007 [6,21,22]. 

Whereas the other tested camphor degradation pathway enzymes were detectible in the first sample taken 15 min after camphor addition to the test medium, it was noticeable that no OTEMO activity was recorded in the 15 min sample and only an extremely low titre detected in the following sample taken 30 min after camphor addition (Figure 2A). Thereafter, the differential rate of synthesis of OTEMO followed a similar albeit delayed profile compared to the other four tested enzymes for a further 30 min before then accelerating to an equivalent rate.

Within this sampling timeframe, the 0–60 min data corresponded to the swop from late log phase growth of *P. putida* NCIMB 10007 at the expense of the progressively diminishing level of succinate to early log phase growth at the expense of (*rac*)-camphor and the 75–135 min data the subsequent exclusively (*rac*)-camphor-dependent mid log phase of growth. Significantly, there was no detectible activity for any of the tested enzymes in the 0 min sample taken immediately post (*rac*)-camphor addition, confirming the absence of these particular enzymes in cells of *P. putida* NCIMB 10007 pre-grown exclusively on succinate as the sole carbon source. The level of succinate remaining in the 0 min samples had fallen to <0.45 mM and this then progressively declined further to be below detectible levels by 75 min, which corresponded with the culture entering mid log phase growth exclusively at the expense of (*rac*)-camphor.

Concomitant with these increases in the titres of the five tested enzymes, the residual level of (*rac*)-camphor in the medium progressively decreased from 5.0 mM at 0 min to >0.3 mM in the 135 min sample. Very similar low levels (0.2 mM) of both 5-*exo*- and 3-*exo*-hydroxycamphor were first detected in the 15 min sample and thereafter remained at similar or slightly higher levels in all further samples taken up to and including 105 min, before subsequently dropping below detectible levels (Figure 2B). The detected presence of the 2,5- and 3,6-DKC enantiomers followed similar profiles and concentrations to those recorded with the enantiomeric *exo*-hydroxycamphors. However, compared to both the *exo*-hydroxycamphor and DKC enantiomers, a consistently higher level of OTE was recorded in each of the tested samples, quickly rising to a concentration of 0.8 mM in the 30 min sample before plateauing at 0.9 mM thereafter up to and including the 135 min sample, at which point the culture of *P. putida* NCIMB 10007 was approaching the end of mid log phase of camphor-dependent growth.

In strict contract, a significant titre of chromosome-coded total ferric reductase activity (Frp1 plus Frp2) was already present in the pre-addition −30 min, −15 min samples and the subsequent 0 min sample taken immediately after (*rac*)-camphor addition: thereafter, the titre maintained a constant relatively low differential rate of synthesis throughout the on-going 135 min sampling timeframe (Figure 2B). Conversely, there was no detectible presence of the chromosome-coded FR Fred in any of the samples taken both before and throughout the 135 min after (*rac*)-camphor addition. However, if the culture was allowed to grow on past 135 min, then a very low level of activity for Fred could be detected in subsequent samples taken at 180 min and 210 min after (*rac*)-camphor was added to the medium, which corresponded with the progression of the culture through late log and into stationary phase growth [23]. This outcome was consistent with previous observations regarding the initial appearance of Fred in (*rac*)-camphor-grown *P. putida* NCIMB 10007 [8]. 

With the exception of the total ferric reductase (Frp1 plus Frp2) titre, succinate-grown cells monitored as a control throughout growth for 135 min after the addition of 5 mM succinate instead of (*rac*)-camphor exhibited none of the above relative changes in the levels of either assayed enzymes or known intermediates of the camphor degradation pathway. An almost identical set of all the above outcomes was also recorded when 5 mM (*rac*)-camphor was added as the inducer-substrate to an equivalent 2.5 mM glutamate-based minimal medium [23].

When the protocol was repeated but with the co-addition of 2 μM rifampicin along with 5 mM (*rac*)-camphor to a succinate-grown culture of *P. putida* NCIMB 10007, no titres for cytP450MO, 5-*exo*-HOcamDH, 2,5-DKCMO, 3,6-DKCMO, OTEMO or Fred were detected in any of the 0–135 min samples and the residual level of camphor in the medium remained at approximately 5 mM throughout (data not shown). Replacing rifampicin with 5 μM actinomycin D resulted in the same total absence of any of either the tested enzyme activities or camphor metabolites. Following the addition of either antibiotic, the differential rate of synthesis of the already present ferric reductases Frp1 and Frp2 initially decreased progressively to approximately 60% of the value recorded prior to the addition of either antibiotic before stabilising in all post-75 min samples [23].

### 3.2. Differential Rates of Synthesis of Key Enzyme Activities Induced by Camphor Pathway Intermediates

Prior preliminary studies [8,9,10] have indicated that relaxed inducer specificity allied to product feedback control may be capable of influencing the titres of some of the early enzymes of the camphor degradation pathway. Consequently, in order to determine whether the established pathway intermediates 2,5-DKC, 3,6-DKC and OTE were themselves capable of serving as inducer-substrates for any of the tested pathway enzymes, sufficient quantities of these three metabolites were first extracted and then purified from large aliquots of spent camphor-based minimal medium using a modification [10] of the original acid-base methods developed by Gunsalus et al. [1].

**Figure 3:**
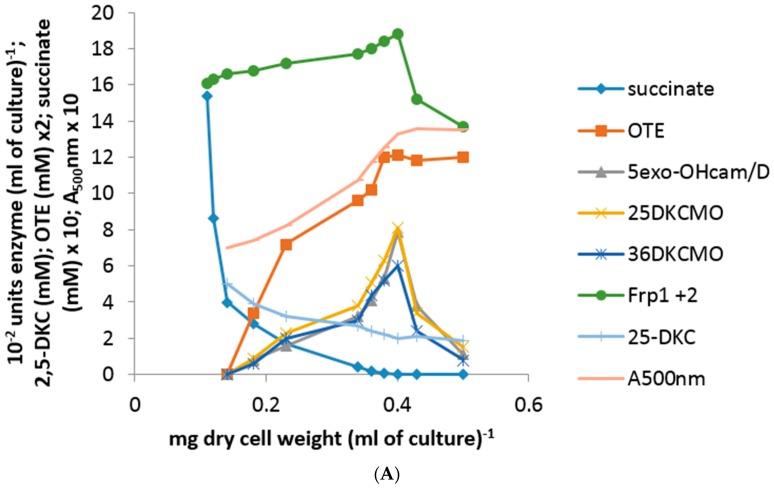

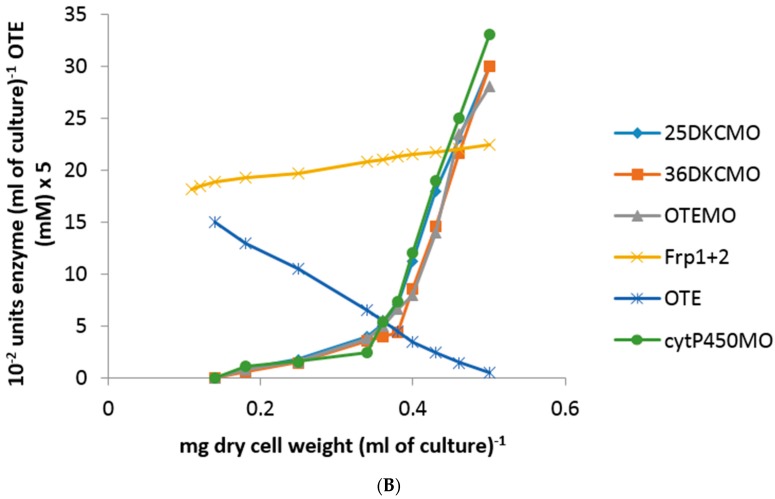
(**A**) Changes in the optical density (A_500_nm) of *P. putida* NCIMB 10007 following addition of 2,5-DKC as inducer-substrate to succinate minimal medium and concomitant changes in the activity of key enzymes and metabolic intermediates of the camphor degradation pathway. An almost identical pattern of changes was recorded when 3,6-DKC was substituted for 2,5-DKC [23]; (**B**) Changes in the optical density (A_500_nm) of *P. putida* NCIMB 10007 following addition of OTE as inducer-substrate to succinate minimal medium and concomitant changes in the activity of key enzymes and metabolic intermediates of the camphor degradation pathway.

The use of either pure 2,5- or 3,6-DKC enantiomer added separately as an inducer-substrate (5 mM) to a succinate-grown culture of *P. putida* NCIMB 10007 resulted in almost identical patterns of progressively accelerating differential rates of synthesis of 5-*exo*-HOcamDH, 2,5-DKCMO and 3,6-DKCMO, along with concomitant increases in the relevant A_500_nm readings, throughout the initial 75 min after the additions occurred (Figure 3A = outcomes following 2,5-DKC addition, equivalent data for 3,6-DKC not intended for publication). However, in strict contrast to the equivalent outcomes recorded with (*rac*)-camphor (*vide infra*), no equivalent titres for cytoP450MO or OTEMO were detected during this initial post-addition phase. Because OTEMO was not induced by either DKC antipode, the OTE product resulting from the induced DKCMOs could not be further metabolised and thus progressively accumulated in the growth medium to approximately 6 mM by 90 min after addition of either DKC enantiomer. OTE at this level has been shown to be toxic to *P. putida* NCIMB 10007 [10] and this combined with the progressive depletion of succinate to below detectible levels in the medium within the same timeframe (Figure 3A) resulted in the consequential cessation of growth as evident both by the plateauing of the post-90 min A_500_nm readings and the stabilization of the level of residual DKC level remaining in the medium. Thereafter, there was a subsequent rapid decline in the titres of the previously induced camphor degradation pathway enzymes in the cultures, probably resulting from starvation-triggered protein turnover [24]. 

Throughout, there was no detectible presence of Fred and the relatively low differential rate of synthesis of the already present Frp1 plus Frp2 titre remained constant both before and after the addition of either DKC enantiomer to the succinate-grown cells until growth ceased at 90 min, after which starvation-induced protein turnover promoted a sharp decline. Significantly, none of the above changes to any of the induced enzyme titres occurred in a culture co-exposed to either DKC enantiomer plus either 2 μM rifampicin or 5 μM actinomycin D [23]. Because of the recognized toxicity of OTE to *P. putida* NCIMB 10007, the corresponding protocol to test the effects of OTE as an inducer-substrate was conducted at a reduced concentration using a 3 mM supplemented succinate minimal medium. The outcomes (Figure 3B) confirmed progressively accelerating differential rates of synthesis of cytP450MO, 2,5-DKCMO, 3,6-DKCMO and OTEMO but noticeably no equivalent titre for 5-*exo*-HOcamDH. Also, most significantly, a detectible titre of induced OTEMO similar to that of the other detected enzymes was first recorded in the initial 15 min sample and thereafter all four detected enzyme titres increased in step throughout the subsequent 120 min sampling schedule, an outcome which contrasted markedly both with the delayed induction profile for OTEMO recorded when using (*rac*)-camphor as the inducer substrate (*vide infra*, Figure 2A) and the total absence of this enzyme when using either DKC enantiomer as the inducer-substrate (Figure 3A). Consistent with its dual role as a growth substrate as well as an inducer, the residual level of the introduced OTE progressively decreased from 3 mM to barely detectible levels by 135 min after addition.

The relatively low differential rate of synthesis of the already present Frp1 plus Frp2 titre remained constant both before and after the addition of OTE to the succinate-grown cells and a very low titre of Fred was only detected in two additional late sample taken 180 min and 210 min after OTE introduction to the medium. As with all the other tested inducer-substrates, none of these changes occurred in a culture co-exposed to OTE plus either 2 μM rifampicin or 5 μM actinomycin D.

### 3.3. The Influence of Isobutyryl-CoA on the Differential Rates of Synthesis of Key (Rac)-Camphor Induced Enzyme Activities

It has been reported that ∆^2,5^-3,4,4-trimethylpimelyl-CoA, the indirect product of OTEMO and the first aliphatic intermediate in the camphor degradation pathway (Figure 1), is further metabolised by a sequence of poorly characterised steps to isobutyryl-CoA [5,25]. Isobutyryl-CoA is a metabolite also generated as an intermediate in the degradation pathways of both pantothenate and the amino acid valine in pseudomonads [7]. As such, it represents a convergent branch point in catabolism in these bacteria. Although poorly characterised, some preliminary studies have suggested that excess isobutyryl-CoA can serve to influence the activity of OTEMO in camphor-grown *P. putida* NCIMB 10007 either by acting as a feedback inhibitor and/or feedback corepressor [9,15,26].

**Figure 4:**
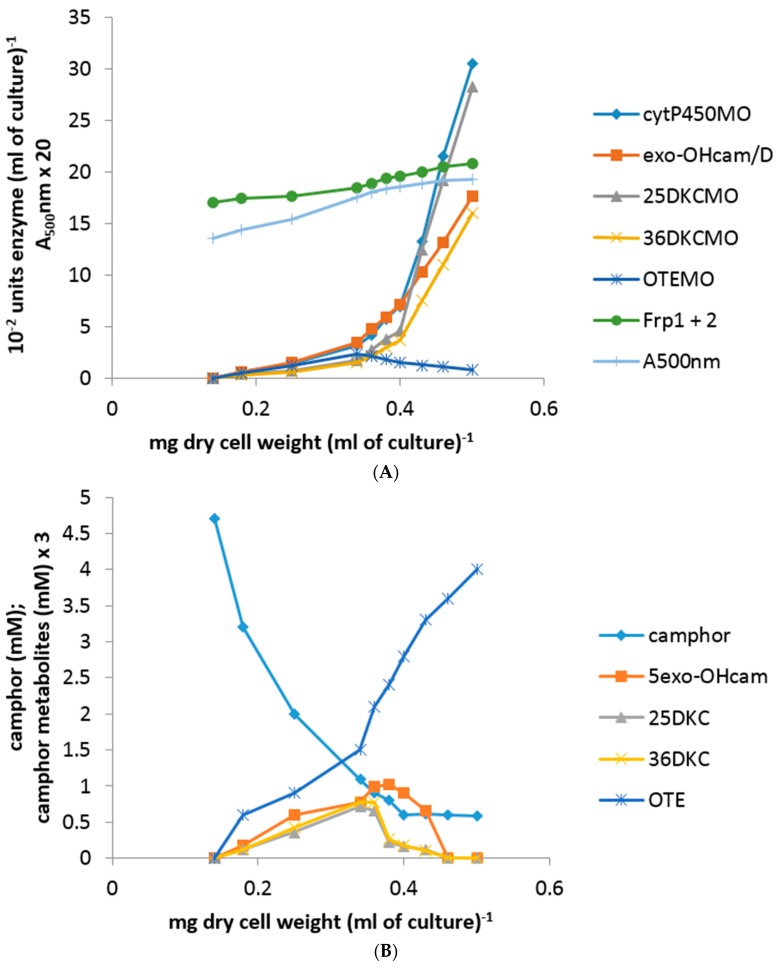
(**A**,**B**) Changes in the optical density (A_500_nm) of *P. putida* NCIMB 10007 following the sequential addition of (*rac*)-camphor and isobutyryl-CoA (+60 min) to succinate minimal medium and concomitant changes in the activity of key enzymes and metabolic intermediates of the camphor degradation pathway.

Following the addition of 5 mM (*rac*)-camphor to a culture of *P. putida* NCIMB 10007 growing on 2.5 mM succinate minimal medium by the standard procedure, the culture was subsequently monitored to establish both the differential rates of synthesis of cytP450MO, 5-*exo*-HOcamDH, 2,5-DKCMO, 3,6-DKCMO and OTEMO: additionally, the residual levels of (*rac*)-camphor, 5-*exo*-hydroxycamphor, 2,5- and 3,6-DKC and OTE present in the medium were recorded. After 60 min, a subsequent addition of 0.05 mM isobutyryl-CoA was made to the growing culture and all the established parameters monitored for a further 75 min. It was notable that both the differential rates of synthesis of all tested enzymes except OTEMO and all assayed compounds except OTE were only marginally influenced by the introduction of 0.05 mM isobutyryl-CoA (Figure 4A vs. the corresponding time courses shown in Figure 2A,B). In strict contrast, the differential rate of synthesis of OTEMO declined progressively throughout the 75 min following the addition of the CoA ester to the growth medium, indicative of the cessation of *de novo* synthesis of the enzyme following addition of the CoA ester to the growth medium, corresponding to repression of the *camG* gene on the CAM-plasmid. Concomitant with the decline in the differential rate of synthesis of OTEMO, there was also a significant corresponding increase in the level of OTE accumulating in the medium (Figure 4B), suggesting that the added CoA ester was also inhibiting the pre-existing titre of OTEMO to some extent. Although the progressively increasing level of accumulating OTE did not exceed the level (>6 mM) at which this camphor metabolite has been reported to be toxic to *P. putida* NCIMB 10007 [10], both the on-going A_500_nm readings (Figure 4A) and the plateauing of the residual camphor level in the medium (Figure 4B) indicated that growth of the culture was significantly curtailed after addition of the isobutyryl-CoA, supporting the proposal that the CoA-ester serves as an effective inhibitor of extant OTEMO activity, thereby precluding access of OTE and thereby most significantly the growth substrate (*rac*)-camphor, to the central pathways of metabolism.

### 3.4. Differential Rates of Synthesis of Key Enzyme Activities when Growing Anaerobically 

Because *P. putida* is a denitrifying species [27], NCIMB 10007 will grow under anaerobic conditions on 2.5 mM succinate minimal medium supplemented with 25 mM NaNO_3_. Precedents recorded with other oxygen-dependent aspects of the metabolism of pseudomonads [28] suggest it may be possible to exploit this biochemical attribute to help distinguish between those observed outcomes of substrate-dependent induction by (*rac*)-camphor and product feedback induction by OTE.

**Figure 5:**
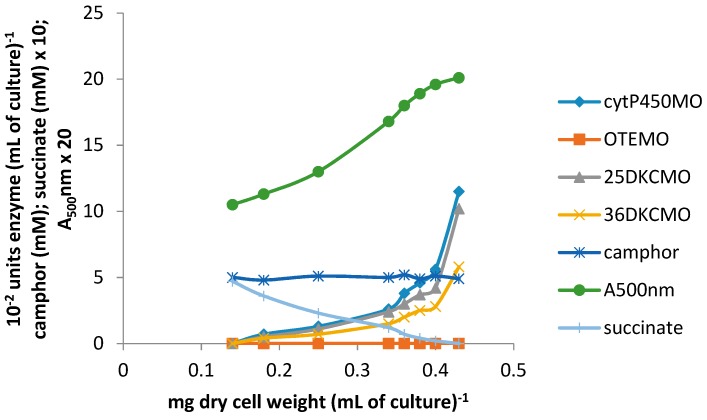
Changes in the optical density (A_500_nm) of a culture of *P. putida* NCIMB 10007 growing anaerobically following addition of (*rac*)-camphor as inducer-substrate to succinate minimal medium supplemented with NaNO_3_ and concomitant changes in the activity of key enzymes and metabolic intermediates of the camphor degradation pathway.

Subsequent to (*rac*)-camphor addition (5 mM) to a culture of *P. putida* NCIMB 10007 growing anaerobically, 0–105 min timed samples were removed via the rubber septum. For each sample, a small aliquot was removed to be assayed as soon as possible for both residual (*rac*)-camphor and various camphor metabolites, while the remainder of the sample was immediately purged with filtered air for 15 min to establish aerobic conditions. The biomass in the air-purged medium was then harvested, relevant cell-free extracts prepared and the various extracts assayed for cytP450MO, the isoenzymic 2,5- and 3,6-DKCMOs and OTEMO by the standard aerobic protocols. Significantly, while the residual level of succinate progressively diminished to below detectible levels in the 0–105 min samples, the level of (*rac*)-camphor in all the tested samples remained unchanged at the addition level of 5 mM (Figure 5), a combination of circumstances which curtailed any further growth of the culture beyond 105 min. Additionally, no detectible trace was found of either 5-*exo*- or 3-*exo*-hydroxycamphor, 2,5- or 3,6-DKC, or OTE [23]. However, progressively increasing titres of cytP450MO and both enantiocomplementary DKCMOs were confirmed in the extracts prepared from all the 15–105 min culture samples that had been subjected to thorough purging with filtered air after harvesting. For all three enzyme activities, the differential rate of synthesis was 80–85% of that recorded in equivalent extracts generated from (*rac*)-camphor-induced cells grown throughout under fully aerobic conditions (Figure 5 vs. Figure 2A). Conversely and most significantly, there was no detectible titre of OTEMO in any of the extracts prepared from the 0–105 min culture samples subjected to thorough post-harvest purging with filtered air. 

### 3.5. Range of Camphor-Related Compounds Able to Support Growth and Act as Inducer-Substrates for Cytochrome P450 Monooxygenase 

The ability of purified samples of the separate (+)- and (−)-camphor enantiomers and various established camphor degradation pathway intermediates (5-*exo*-hydroxycamphor, 3-*exo*-hydroxy-camphor, 2,5-DKC, 3,5-DKC and OTE), plus a number of other compounds structurally related to either bicyclic camphor or monocyclic OTE, were tested both for the dual ability to act as inducer-substrates for cytP450MO and to support growth of *P. putida* NCIMB 10007. The 17-keto steroids androsterone, androstenedione and oestrone were also screened because this type of molecule was originally reported to induce camphor degradation pathway enzymes in *P. putida* NCIMB 10007 [2], although this claim was subsequently retracted [10]. 

All the compounds were included at 3 mM as the sole organic carbon source in aliquots of the standard mineral salts medium with the exception of the three 17-keto steroids which were tested at a lower concentration (2 mM) because of associated solubility issues. Optical density readings (A_500_nm) were followed to confirm any resultant growth and that data used for each competent carbon source to establish the corresponding mid log phase point of growth, at which point the corresponding cytP450MO activity was determined as the titre of the enzyme has been found to be maximal at this point in *P. putida* NCIMB 10007 growing at the expense of (*rac*)-camphor [8].

All of the established camphor degradation pathway intermediates, plus the highly purified (+)- and (−)-camphor enantiomers, proved to be competent sole growth substrates when included at 3 mM in the standard minimal medium and induced similar titres of cytP450MO at their respective mid log phase point of growth (Supplementary Materials Table S1). However, a number of other tested compounds that are structurally related to a greater or lesser extent to camphor failed to support any detectible growth when tested as the sole added carbon source. This included compounds both more (fenchone, 2,3-bornadione) and less (1,8-cineole, nopinone, 3-methylene-2-norbornanone, 2-norbornanone, bornane, bicyclo[3.2.0]hept-2-en-6-one, 2,4,4-tri-methylcyclopentanone, 2,2,4-trimethylcyclopentanone, 2-*n*-hexylcyclopentanone, 2-pentylcyclo-pentanone, 3-methylcyclopentanone, 3-methylcyclopent-2-enone, 2-methylcyclopentanone) highly substituted than camphor. In addition, all three of the 17-keto steroids with D-ring conformations that show some structural resemblance to camphor [2] failed to serve as growth substrates. 

**Figure 6:**
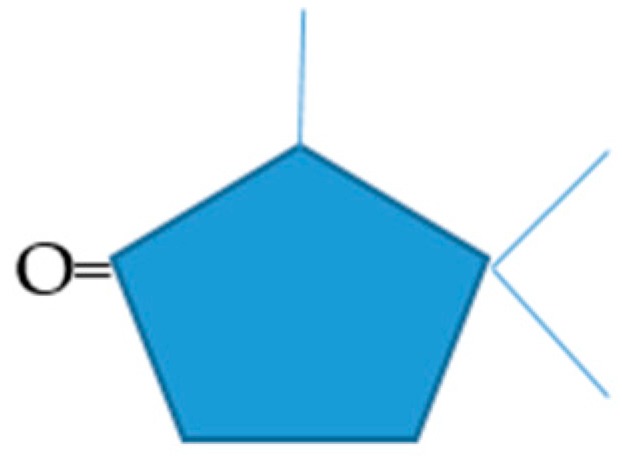
Default carbocyclic structural features that delineate efficacy as a growth substrates for *Pseudomonas putida* NCIMB 10007. Both the number and positions of the three methyl/methylene bridge substituents relative to the carbonyl group are critical structural determinants of functionality.

These various outcomes suggest that the default structural features for efficacy as a growth substrate, which in turn implies competence to serve either directly or indirectly as an inducer-substrate for a complete suite of relevant enzymes necessary to ensure entry into the central pathways of metabolism, is a core pentacyclic ketone ring substituted with a definitive pattern of three methyl/methylene bridge groups (Figure 6). Prior studies have shown that some of the compounds which failed to support growth (2,3-bornadione, norbornanone, bicyclo[3.2.0]hept-2-en-6-one, 2-*n*-hexyl- and 2-pentylcyclopentanone) do undergo lactonization with purified preparations of relevant monooxygenases from (*rac*)-camphor-grown *P. putida* NCIMB 10007 [5,16,21,22,29] but clearly that is insufficient to guarantee the role of a sole carbon source for growth.

## 4. Discussion

**Figure 7:**
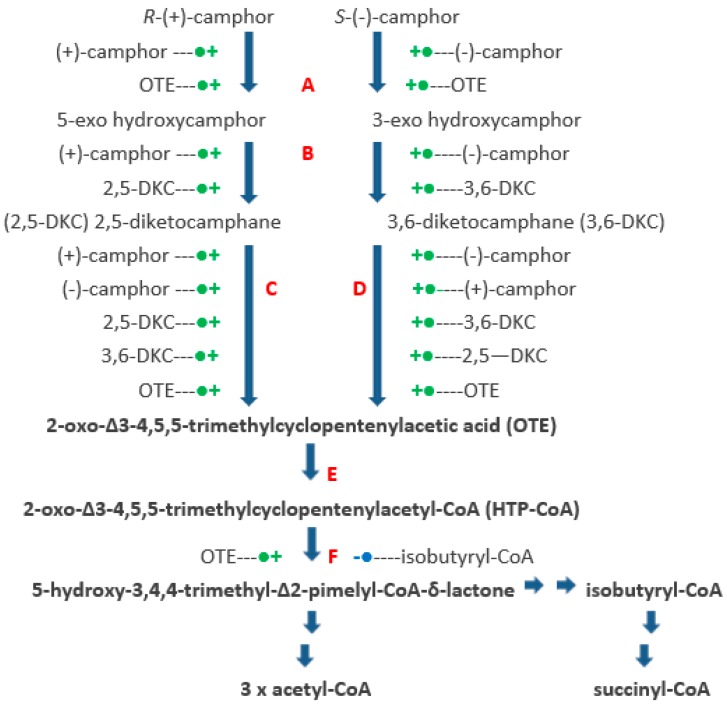
Transcriptional controls of the pathway of (+)- and (−)-camphor degradation in *P. putida* NCIMB 10007. ----●**+** = induction: **-**●---- = repression: A = cytochrome P450 monooxygenase (*camCAB*): B = exo-hydroxycamphor dehydrogenase (*camD*): C = 2,5-diketocamphane 1,2-monooxygenase (*camE_25-1_* + *camE_25-2_*): D = 3,6-diketocamphane 1,6-monooxygenase (*camE_36_*): E = 2-oxo-∆^3^-4,5,5-trimethylcyclopentenylacetyl-CoA synthetase (*camF1 + F2*): F = 2-oxo-∆^3^-4,5,5-trimethylcyclopentenylacetyl-CoA monooxygenase (*camG*).

Although two earlier preliminary studies of the initial enzymes of the camphor degradation pathway in *P. putida* NCIMB 10007 obtained some limited evidence for elements of regulatory controls without establishing any relevant molecular mode(s) of action [9,10], the presented data characterise for the first time induction profiles of all the key oxygen-dependent enzymes involved in the catabolism of bicyclic (*rac*)-camphor to the first aliphatic intermediate, ∆^2,5^-3,4,4-trimethylpimelyl-CoA. In each case the efficacy of both the relevant substrate and pathway metabolites as inducers has been established (Figure 7). Further, inhibitors with characterised functionality have been used to identify the mechanistic basis for the pertinent regulatory controls. In addition to the separate camphor antipodes, only compounds which are recognised camphor degradation pathway intermediates were found to be viable growth substrates for *P. putida* NCIMB 10007, an outcome similar to that recorded by a comprehensive study of mandelate degradation by *P. putida* A.3.12 [29].

With the exception of the two ferric reductases Frp1 and Frp2, for which there was an already pre-existing titre in the succinate-grown cells used to monitor the inductive effects of (*rac*)-camphor and its various metabolites, it was noticeable that all the assayed enzymes exhibited an initial nonlinear differential rate of synthesis during early log phase growth at the expense of each of the tested inducer-substrates. A similar pattern of nonlinear differential rate of synthesis characterised the induction of β-galactosidase in *Escherichia coli* by relevant inducers, which proved to be due to the time required to co-induce a corresponding permease [30,31]. However, no evidence has ever been presented for the existence of a relevant permease in camphor-grown *P. putida* NCIMB 10007. A more likely alternative possibility is that in each case the initial nonlinear portion of the plot reflects release from some form of catabolite repression promoted by the progressively declining low level of remaining succinate in the growth medium throughout the short period of time (approximately 60 min) that followed the addition of an inducer-substrate. Prior studies have shown that succinate can cause catabolite repression of 5-*exo*-HOcamDH in (*rac*)-camphor-induced *P. putida* NCIMB 10007 [9,10], although the effective levels reported were an order of magnitude higher than those relevant to the current studies, which were approximately 0.45 mM at the time that the tested inducers were added to on-growing cultures. However, it is highly significant that as growth of *P. putida* NCIMB 10007 progressed through the subsequent short phase of transitional growth that followed the introduction of each inducer-substrate, the already low level of succinate in the medium continued to fall until it was below detectible levels after 75–90 min and this consistently corresponds with the time after which the differential rates of synthesis of each of the induced enzymes increased to their maximum recorded rate.

The results obtained from using (*rac*)-camphor as the added effector are consistent with cytP450MO, 5-*exo*-HOcamDH (which is equally active with 3-*exo*-hydroxycamphor [10]) and the enantiocomplementary 2,5- and 3,6-DKCMOs but significantly not OTEMO (*vide supra*), being subject to coordinate induction [11,32] by (*rac*)-camphor itself within the first few minutes of its introduction into the growth medium. It is known [33] that the CAM-plasmid genes coding for cytP4540MO (*camCAB*) and 5-*exo*HOcamDH (*camD*) constitute a single polycistronic operon (*camRDCAB*), the expression of which is controlled by *camR*, the concatenated regulator gene but the genes coding for the isoenzymic DKCMOs are more distally located on the plasmid [5]. As camphor is the initial substrate of the degradative pathway and these particular enzymes catalyse the first three successive steps of the camphor degradation pathway, this form of initial substrate-dependent control that has been termed ‘from the top’ coordinate pathway regulation and is a characteristic feature of a number of other catabolic pathways in pseudomonads [34,35].

The particular effects promoted by the use of (*rac*)-camphor as inducer-substrate on the enantiocomplementary DKCMOs are interesting for several reasons. Firstly, these enzymes are FNR-dependent fd-TCMOs able to source FNR not only from chromosome-coded enzymes (Frp1 and 2 and Fred) but alternatively CAM-plasmid coded PdR, a protein which additional serves a role as a functioning subunit of cytP450MO [8]. Taking the titre of trimeric cytP450MO as being a reflexion of the activity of its PdR subunit, then the activity profile of this 46.5 kDa redox intermediate and also that of the combined ferric reductases (Frp1 plus Frp2) are both entirely consistent with their ability to support FNR-dependent DKCMO activity throughout the early– and mid–log phases of growth on (*rac*)-camphor. Further, the absence of any detectible titre of Fred until 180–210 min, at which point the residual (*rac*)-camphor in the medium has fallen to barely detectible levels and the culture was progressing from late log into stationary phase growth, reinforces the proposal [8] that Fred plays no significant role in trophophasic growth and is more likely to be induced specifically to support one or more aspects of secondary metabolism in *P. putida* NCIMB 10007. It is known that strains of *P. putida* initiate the synthesis of a suite of novel secondary metabolites on entering idiophase [36] and a recent survey of 58 strains revealed biosynthetic gene clusters for a wide range of nonribosomal peptides, polyketides and bacteriocins [37]. 

Secondly, it is significant that compared to 2,5-DKCMO, the differential rate of synthesis of 3,6-DKCMO is slower and consequentially the equivalent titres at corresponding times lower, throughout the early– and mid–log phases of growth. The outcomes from these differential plots of the relevant key regulatory events substantiate some previous more conventional preliminary studies of *P. putida* NCIMB 10007 using either (*rac*)-camphor or highly purified samples of the separate (+)- or (−)-camphor enantiomers as a carbon source [6,11,21,22,38] in identifying not only equivalent differences in relative levels of induction for the two DKCMOs but also confirmed that each DKCMO was not only induced by growth on the camphor enantiomer from the corresponding enantiomeric series ((+)-camphor = 2,5-DKCMO: (−)-camphor = 3,6-DKCMO) but was also ‘cross induced’ by growth on the complementary camphor enantiomer. This type of relaxed inducer specificity has been advanced as being a major contributor to the eclectic form of regulatory control that characterises (*rac*)-camphor-grown *P. putida* NCIMB 10007 and which is a characteristic feature of a number of other degradative pathways in various pseudomonads [20,34,35].

The effect promoted when using (*rac*)-camphor as inducer-substrate on OTEMO, which catalyses the fourth enzyme-catalysed step in the camphor degradation pathway, however, indicates a significantly different element of regulatory control other than coordinate induction ‘from the top’ of the pathway. Characteristically, no detectible OTEMO activity is apparent until the very low titre recorded in the sample taken 30 min after the addition of (*rac*)-camphor. During that hiatus, OTE produced by the concerted action of the already co-ordinately induced enzymes catalysing the first three steps of the degradative pathway progressively accumulates in the growth medium to a level of approximately 0.8 mM. This threshold level of OTE is sufficient to prompt the monocyclic metabolite to then serve as the inducer-substrate for OTEMO, hence triggering its delayed appearance relative to the earlier enzymes of the camphor degradation pathway. This OTE-triggered action in turn serves to stabilise the level of OTE accumulating in the growth medium. This form of control, which in this case involves the initial metabolism of (*rac*)-camphor to OTE prior to this monocyclic pathway intermediate then itself serving as the inducer-substrate for OTEMO, has been termed ‘sequential induction’ or ‘indirect substrate induction’ and like ‘from the top’ coordinate induction (*vide infra*), is widely encountered in catabolic pathways in pseudomonads [34,39,40]. The consequential nature of ‘indirect substrate induction’ also contributes significantly to interpreting the outcomes from the corresponding (*rac*)-camphor induction study conducted under anaerobic growth conditions. Thus, whereas by deploying a suitable post-sampling aerobic assay procedure it is possible to confirm that (*rac*)-camphor had progressively induced titres of cytP450MO and both enantiocomplementary DKCMOs in the 15–105 min samples of *P. putida* NCIMB 10007 grown anaerobically in argon-flushed NO_3_^−^-supplemented minimal medium, those induced oxygen-dependent enzymes are unable to function in situ in the growing culture because of the prevailing anaerobic conditions in the medium. Anaerobic growth therefore occurs exclusively at the expense of residual succinate, with no metabolism of the added (*rac*)-camphor, as the corresponding analyses confirm. Consequently, no OTE is generated in the growing cells and hence the induction of OTEMO is not initiated, as confirmed by the absence of a detectible OTEMO titre when such anaerobically grown cells are subsequently tested aerobically. These outcomes therefore reinforce the role of OTE generated from (*rac*)-camphor as the ‘indirect substrate inducer’ in the sequential induction of OTEMO in *P. putida* NCIMB 10007. Similar comparative anaerobic vs aerobic protocols have been used to confirm equivalent oxygen-dependent elements of regulatory control of other catabolic pathways in pseudomonads [28,41,42].

That all of these recorded regulatory controls triggered by (*rac*)-camphor in *P. putida* NCIMB 10007 involve forms of genetic control effective at the level of transcription of the relevant genes is evident from the clear outcomes of the studies in which 2 μM rifampicin (a potent inhibitor of prokaryotic RNA polymerase) and 5 μM actinomycin D (promotes prokaryotic DNA intercalation) were co-added along with (*rac*)-camphor to late log phase succinate-grown cultures. For cytP450MO (*camA* + *camB* + *camC*), 5-*exo*-HOcamDH (*camD*), the oxygenating subunits of the enantiomeric DKCMOs (*camE_25-1_* + *camE_25-2_* + *camE_36_*) and OTEMO (*camG*), all the relevant genes are located on the CAM-plasmid and although not well characterized, are believed to share elements of control via a number of different plasmid-located transcriptional regulator genes [3,4,5]. Fujita et al. [33] established that the TetR-type *camR* regulator gene located in the polycistronic *camRDCAB* operon codes for a repressor protein that is suppressed by camphor and is thereby able to directly influence the titres of both 5-*exo*-HOcamDH and the three-component cytP45OMO in camphor-grown *P. putida* but they did not report whether (+)-, or (−)-, or (*rac*)-camphor was used as the growth substrate. There are additionally three other plasmid-located TetR-type regulator genes (*orf5* [*camS*], *orf11* [*camU*], *orf20* [*camV*]) and one other LysR-type regulator gene (*orf7* [*camT*]), although the individual role(s) of the corresponding repressor proteins and whether they also exhibit any additional cooperative function(s) is currently uncharacterised. One possible involvement could be in providing the transcription-based mode of action implicit in the recorded action of isobutyryl-CoA, which when tested at the relatively low level of 0.05 mM proved to be a very effective feedback corepressor of (*rac)*-camphor-induced OTEMO (*camG* [*orf3*]) in *P. putida* NCIMB 10007. 

The suite of separate studies undertaken with the purified recovered samples of the pathway intermediates OTE, 2,5- and 3,6-DKC added as substrate-inducers itself reveal additional elements of inductive regulatory control effective at the level of transcription as confirmed by the corresponding comprehensive inhibitory action of both rifampicin and actinomycin D. OTE, one of the last alicyclic intermediates in the (*rac*)-camphor degradation pathway, although toxic to *P. putida* NCIMB 10007 at >6 mM [10], proved to be effective as an inducer-substrate when added to succinate minimal medium at 3 mM. Used at this level, OTE promotes coordinate induction of not only of OTEMO, the enzyme for which it is a substrate as well as an inducer but also the three other monooxygenases (cytP450MO, 2,5-DKCMO and 3,6-DKCMO) that precede OTEMO in the degradation pathway and that thus serve key roles in generating OTE from (*rac*)-camphor and both DKC enantiomers if and when they are available. Consequently, unlike the outcome recorded when using (*rac*)-camphor as the inducer, a titre for OTEMO was recorded in the first sample, taken 15 min after OTE addition, because there is no equivalent delay corresponding to the necessity to generate OTE as an ‘indirect substrate inducer’ in the culture. This pattern of regulatory control, resulting in the coordinate induction of cytP450MO, 2,5-DKCMO and 3,6-DKCMO by using 3 mM OTE as the inducer-substrate, has been referred to alternatively as ‘from the bottom’ or ‘product induction’ regulation and has been characterised in various other catabolic pathways both in pseudomonads [34,35,43,44,45,46,47] and certain other bacteria [48,49,50,51]. Product induction is a seemingly counter intuitive regulatory mechanism. However, an evolution-based explanation has been advanced [34], dependent on the proposition that catabolic evolution of a pathway is likely to have proceeded from the ‘bottom’ to ‘top’ by sequential accretion of new units of physiological function, thus enabling novel substrates to be converted to metabolites that were already utilised by established metabolic routes. Superimposed on the acquisition of additional structural genes for enzymes of such a catabolic sequence regulatory genes co-evolved to restrict the expression of the newly acquired structural genes to circumstances in which their function would be effective.

One other outcome from using 3 mM OTE as the inducer-substrate that is particularly interesting is the failure of this camphor metabolite to induce any detectible titre of 5-*exo*-HOcamDH, an outcome which contrasts with the induction of a significant titre of cytP450MO and the other oxygen-dependent ‘early enzymes’ in the camphor degradation pathway. It has been conclusively shown that the corresponding CAM-plasmid gene for the dehydrogenase (*camD*) is concatenated with the *camCAB* genes that code for the three subunits of cytP450MO in the polycistronic *camRDCAB* operon, the collective expression of which is controlled by the *camR* regulator gene [33]. The recorded ability of OTE to induce cytP450MO without the co-induction of 5-*exo*-HOcamDH is indicative of additional as yet uncharacterised elements transcriptional control enabling selective expression of a specific gene (*camD*) within the polycistronic *camRDCAB* operon of *P. putida* NCIMB 10007. An equivalent observation was recorded with an OTE-induced culture by Gunsalus et al. [9], although the significance of this was not recognised and commented on at the time which pre-dated knowledge of the relevant sequence data for the CAM-plasmid by several years.

The highly characteristic biphasic outcomes recorded using purified samples of either of the two DKC enantiomers as inducer-substrates require a more nuanced explanation. Each enantiomer when tested separately as the inducer-substrate initially promotes almost identical patterns of progressively accelerating differential rates of synthesis of 5-*exo*-HOcamDH, 2,5-DKCMO and 3,6-DKCMO but most significantly not cytP450MO or OTEMO for the initial 75 min after the additions occurred, implicating a pattern of selective coordinate induction that included both ‘from the bottom’ as well as conventional substrate-induced regulatory control elements. The equivalent increases in the titres of both the enantiocomplementary DKCMO isoenzymes promoted by each DKC enantiomer when added separately as the inducer-substrate is interesting because it supports previous preliminary studies which hinted at an equivalent element of ‘cross inducibility’ for these enzymes by the two enantiomers of camphor (*vide infra*, [6,13,22,30,38]). The failure of either DKC enantiomer to induce OTEMO results in multiple consequences. Firstly, because OTE could not be further metabolised, it progressively accumulates in the medium, rising after 75 min to approximately 6 mM, a level at which this metabolite is known to be toxic to NCIMB 10007 [10]. This, together with the depletion of succinate to below detectible level by 60–75 min results in a cessation of growth of the relevant culture after 75 min and a concomitant rapid decline in the titres of the limited suite of camphor degradation pathway enzymes that have been induced (5-*exo*-HOcamDH, 2,5-DKCMO and 3,6-DKCMO), most probably a direct outcome of starvation-triggered protein turnover, as is known to occur in non-growing cultures [24,51,52].

Collectively, the presented data characterize for the first time the key initial induction profiles of all the key oxygen-dependent enzymes involved in the catabolism of bicyclic (*rac*)-camphor to the first aliphatic intermediate, ∆^2,5^-3,4,4-trimethylpimelyl-CoA. In each case the efficacy of both the relevant substrate and pathway metabolites as inducers has been established (Figure 7). Further, inhibitors with characterised functionality have been used to identify the mechanistic basis for the pertinent regulatory controls. In addition to the separate camphor enantiomers, only compounds which are recognised camphor degradation pathway intermediates were found to be viable growth substrates for *P. putida* NCIMB 10007, an outcome similar to that recorded by a comprehensive study of mandelate degradation by *P. putida* A.3.12 [53]. A number of the transcription-based controls identified in (*rac*)-camphor-grown *P. putida* NCIMB 10007 signal relaxed specificities of the relevant repressor proteins involved. These include examples of ‘forward induction’ (the DKCMO isoenzymes by camphor as none of the relevant genes are not concatenated to the *camRDCAB* polycistronic operon [*vide infra*]), ‘back induction’ (cytP450MO and the DKCMOs by OTE) and ‘cross induction’ (the 2,5- and 3,6-DKCMOs by the corresponding bornadione substrate from the opposite enantiomeric series). In addition, the enzymes *exo*-hydroxycamphor dehydrogenase [10], both enantiocomplementary DKCMOs [5,13,21,22] and OTEMO [16] have been demonstrated to be effective with a number of alternatives substrates, indicating that these catalytically active proteins also have relaxed substrate specificities. For the dehydrogenase, this catalytic tolerance embraces both complementary epimeric and stereoisomeric substrate compatibility, while for the DKCMOs, recombinant forms of the enzymes have been confirmed to be active with both enantiomers from the camphor and DKC isomeric series [54,55]. While the data presented in Table S1 suggest that the latitude of tolerated variance of some of these relaxed specificities may be relatively narrow, the ‘molecular promiscuity’ of the various relevant repressor proteins and enzymes coded for by the CAM-plasmid of *P. putida* NCIMB 10007 that these and the cited related prior studies have established is none the less impressive. Equivalent broad specificity-based controls have been characterised in various pseudomonads, including *P. putida* A.3.12, that can catabolise a variety of aromatic compounds via the *β*-ketoadipate pathway [35,42,56,57,58,59].

Although not as well characterised at the time, it was proposed by Irwin Gunsalus (the founding father of microbial camphor biochemistry) that broad specificity of both induction (repressor proteins) and catalysis (enzymes) could either represent a way of vesting *P. putida* NCIMB 10007 with considerable metabolic flexibility [2], or alternatively [10] that it may represent the cost, or possibly result, of possessing genetic information (the CAM-plasmid) for a degree of specialization, namely the degradation of a structurally unique compound such as camphor but that the relative rarity of camphor in the biosphere may minimise unnecessary enzyme synthesis.

## Supplementary Materials

The following are available online at http://www.mdpi.com/2076-2607/6/2/41/s1, Table S1: Growth (maximum A500_nm_) of *P. putida* NCIMB 10007 and resultant cytochrome P450 monooxygenase titre achieved following inoculation into minimal salts medium supplemented with various bicyclic and monocyclic compounds used as sole carbon source.
