# Supplementary material for: Regulation of Camphor Metabolism: Induction and Repression of Relevant Monooxygenases in Pseudomonas putida NCIMB 10007
Source: Microorganisms. 2018 May 7;6(2):41. doi: 10.3390/microorganisms6020041 (PMC6027186; doi:10.3390/microorganisms6020041)
Supplement: Supplementary file 1 [file microorganisms-06-00041-s001.pdf]

Supplementary Materials

Table S1

Test substrate added as sole carbon source	Max A_{500}nm reading achieved (time after substrate addition)	Max cytP450MO titre achieved Enzyme units (mg protein^{-1}) x 10^3 (time after substrate addition)
(+)-camphor	0.43 (190 min)	62 (130 min)
(-)-camphor	0.38 (200 min)	44 (120 min)
5-*exo* hydroxycamphor	0.40 (200 min)	54 (130 min)
3-*exo* hydroxycamphor	0.36 (200 min)	46 (140 min)
2,5-diketocamphane	0.45 (180 min)	60 (120 min)
3,6-diketocamphane	0.41 (190 min)	49 (130 min)
OTE	0.40 (180 min)	55 (120 min)
androsterone	no detectible growth	not assayed
androstenedione	no detectible growth	not assayed
estrone	no detectible growth	not assayed
fenchone	no detectible growth	not assayed
2,3-bornadione	no detectible growth	not assayed
2-norbornanone	no detectible growth	not assayed
3-methylene-2-norbornanone	no detectible growth	not assayed
bicyclo[3.2.0]hept-2-en-6-one	no detectible growth	not assayed
1,8-cineole	no detectible growth	not assayed
nopinone	no detectible growth	not assayed
bornane	no detectible growth	not assayed
norbornane	no detectible growth	not assayed
2,4,4-trimethylcyclopentanone	no detectible growth	not assayed
2,2,4-trimethylcyclopentanone	no detectible growth	not assayed
2-n-hexylcyclopentanone	no detectible growth	not assayed
2-pentylcyclopentanone	no detectible growth	not assayed
3-methylcyclopentanone	no detectible growth	not assayed
3-methylcyclopent-2-enone	no detectible growth	not assayed
2-methylcyclopentanone	no detectible growth	not assayed
